# Influence of Halogen Substituents on the Catalytic Oxidation of 2,4,6-Halogenated Phenols by Fe(III)-Tetrakis(*p*-hydroxyphenyl)porphyrins and Potassium Monopersulfate

**DOI:** 10.3390/molecules17010048

**Published:** 2011-12-22

**Authors:** Masami Fukushima, Yusuke Mizutani, Shouhei Maeno, Qianqian Zhu, Hideki Kuramitz, Seiya Nagao

**Affiliations:** 1 Laboratory of Chemical Resources, Division of Sustainable Resources Engineering, Graduate School of Engineering, Hokkaido University, Sapporo 060-8628, Japan; 2 Environmental Energy and Science, Graduate School of Science and Engineering for Research, University of Toyama, Gofuku 3190, Toyama 930-8555, Japan; 3 Low Level Radioactivity Laboratory, Institute of Nature and Environmental Technology, Kanazawa University, Wake, Nomi-shi, Ishikawa 923-1224, Japan

**Keywords:** iron(III)-porphyrin, humic acid, hydroquinone, catalytic oxidation, 2,4,6-halogenated phenols, halogen substituent, electronegativity

## Abstract

The influence of halogen substituents on the catalytic oxidation of 2,4,6-trihalogenated phenols (TrXPs) by iron(III)-porphyrin/KHSO_5_ catalytic systems was investigated. Iron(III)-5,10,15,20-tetrakis(*p*-hydroxyphenyl)porphyrin (FeTHP) and its supported variants were employed, where the supported catalysts were synthesized by introducing FeTHP into hydroquinone-derived humic acids via formaldehyde poly-condensation. F (TrFP), Cl (TrCP), Br (TrBP) and I (TrIP) were examined as halogen substituents for TrXPs. Although the supported catalysts significantly enhanced the degradation and dehalogenation of TrFP and TrCP, the oxidation of TrBP and TrIP was not enhanced, compared to the FeTHP catalytic system. These results indicate that the degree of oxidation of TrXPs is strongly dependent on the types of halogen substituent. The order of dehalogenation levels for halogen substituents in TrXPs was F > Cl > Br > I, consistent with their order of electronegativity. The electronegativity of a halogen substituent affects the nucleophilicity of the carbon to which it is attached. The levels of oxidation products in the reaction mixtures were analyzed by GC/MS after extraction with *n*-hexane. The most abundant dimer product from TrFP via 2,6-difluoroquinone is consistent with a scenario where TrXP, with a more electronegative halogen substituent, is readily oxidized, while less electronegative halogen substituents are oxidized less readily by iron(III)-porphyrin/KHSO_5_ catalytic systems.

## 1. Introduction

Halogenated phenols, especially chlorophenols and bromophenols, are frequently included in plastics as flame retardants and for wood preservation. They can be leached from electronic equipment and wood wastes in landfills [1]. In addition, it has been shown that some halogenated phenols function as endocrine disruptors [2] and are listed as priority pollutants by the United States Environmental Protection Agency [3]. Because leachates from wastes in landfills are frequently discharged into natural water systems, the removal of halogenated phenols from leachates is an important issue, in terms of reducing the potential risk of pollution and related health issues.

**Figure 1:**
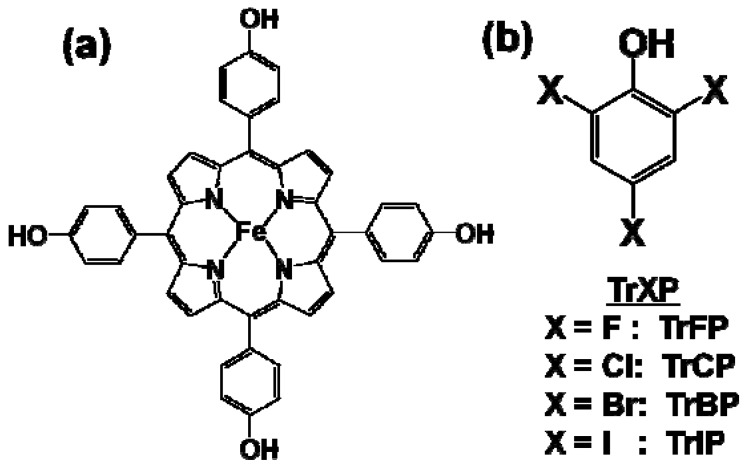
Chemical structures of (**a**) iron(III)-5,10,15,20-tetrakis(*p*-hydroxyphenyl) porphyrin (FeTHP); and (**b**) 2,4,6-trihalogenated phenol (TrXP).

Iron(III)-porphyrin complexes have been regarded as biomimetic models of oxidative enzymes such as ligninases and peroxidases [4], and can catalyze the oxidative dechlorination of chlorophenols in the presence of an oxygen donor such as H_2_O_2_ and KHSO_5_ [5,6,7,8,9,10]. These observations suggest that iron(III)-porphyrins have the potential for applications to clean technologies for the remediation of chlorophenol-contaminated water and soil [11,12,13,14]. However, the self-degradation of iron(III)-porphyrins in the presence of H_2_O_2_ or KHSO_5_ leads to catalyst inactivation [15,16,17]. To suppress catalyst self-degradation, the authors examined the possibility of incorporating iron(III)-5,10,15,20-tetrakis(*p*-hydroxyphenyl)porphyrin (FeTHP, Figure 1a) into phenolic moieties in natural polyphenols such as humic acid (HA), via formaldehyde polycondensation [18,19]. The resulting supported catalyst was found to be more stable to self-degradation and enhanced the degradation of chlorophenols. In addition, the FeTHP that was introduced into *p*-hydroquinone modified HA (HQ-HA-FeTHP) resulted in enhanced catalytic activities for the oxidation of chlorophenol, compared to FeTHP alone and other HA derivatives modified with FeTHP [20].

Some iron(III)-porphyrin catalysts have also been examined for their ability to catalyze the oxidation of bromophenols, which are mainly utilized as brominated flame retardants. In this case, the degree of debromination was much lower than that of the dechlorination for chlorophenols [21,22]. This suggests that the degree of dehalogenation is dependent on the types of substrates rather than on the activities of catalysts. Halogenated phenols, such as fluorophenols and iodophenols, are used as components of liquid crystals. Thus, leachates from TV and PC monitors in landfills are also a concern, in terms of water contamination with halogenated phenols. The dehalogenation that accompanies the oxidation of halogenated phenols is generally believed to be crucial for the detoxification of such materials. Nevertheless, the characteristics of oxidation by the iron(III)-porphyrin catalysts as a function of halogen substituents in halogenated phenol derivatives have not been compared. In the present study, the influence of halogen substituents on the oxidation of 2,4,6-trihalogenated phenols (TrXPs, X = F, Cl, Br, and I, Figure 1b) by FeTHP/KHSO_5_ and HQ-HA-FeTHP/KHSO_5_ catalytic systems were investigated, in terms of degradation rates, the levels of dehalogenation and the oxidation products that are produced.

## 2. Results and Discussion

### 2.1. Influences of Solution pH on the Degradation and Dehalogenation of TrXPs

**Figure 2:**
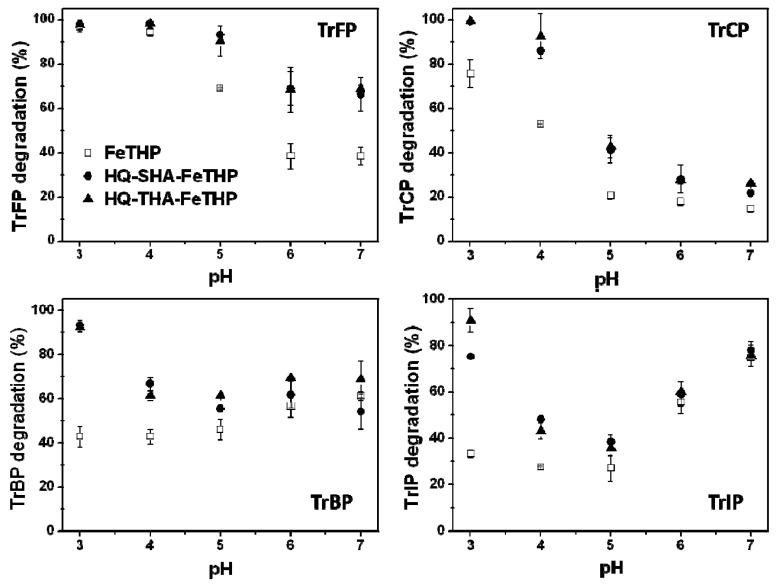
Influence of pH on the percent degradation of TrXP: [Catalyst] 5 μM, [TrXP]_0_ 150 μM, [KHSO_5_] 500 μM.

In the present study, TrXPs were used as organic substrates, and two HQ-HA-FeTHP catalysts (HQ-SHA-FeTHP and HQ-THA-FeTHP) were prepared from two types of HAs as described in the Experimental Section. First, control experiments were conducted at pH 4 in the presence of KHSO_5_ (500 μM) alone and of the catalysts (5 μM) alone. Under these conditions, no TrXP degradation was observed. Figure 2 shows the influence of pH on the percent TrXP degradation. For TrFP and TrCP, the percentages of TrXP degradation increased with decreasing pH. However, for the cases of TrBP and TrIP using the FeTHP catalyst, the percentage of TrXPs was somewhat higher at higher pH, while the percent TrXP degradation using the HQ-HA-FeTHPs reached a minimum at pH 5. 

For all of the HQ-HA-FeTHP catalysts, pH 3 was the maximum pH for degrading TrXPs, and the degradation was significantly enhanced by the HQ-HA-FeTHP catalysts compared to the FeTHP catalyst, except for TrFP. It was reported that the oxidation of TrCP by a water-soluble iron(III)-tetrakis(*p*-sulfonatophenyl) porphyrin/KHSO_5_ system was facilitated at in lower pH [5], which is consistent with the results in Figure 1. 

**Figure 3:**
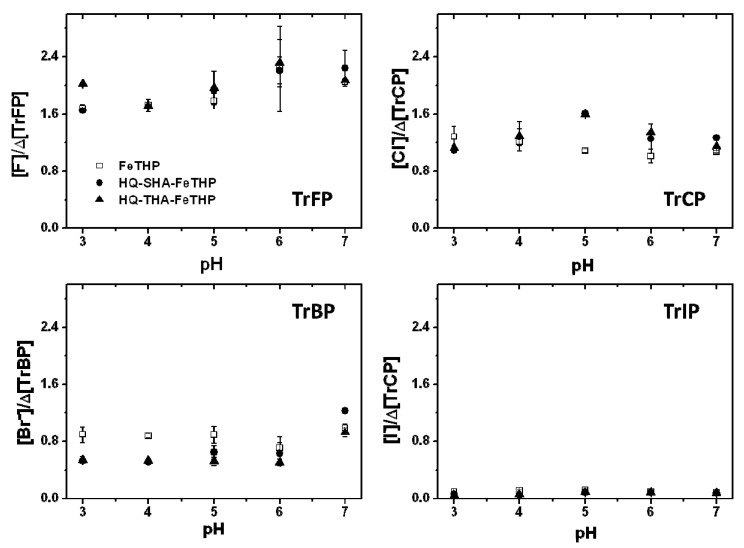
Influence of pH on the number of F^−^, Cl^−^, Br^−^ and I^−^ released as a result of TrXP oxidation: [Catalyst] 5 μM, [TrXP]_0_ 150 μM, [KHSO_5_] 500 μM.

Figure 3 shows the influence of pH on the numbers of halogen atoms released from TrXP during the catalytic oxidation. The numbers of released halogen atoms were calculated as follows:


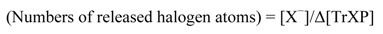
(1)

where [X^−^] and Δ[TrXP] denote the concentrations of halide ions and the subtraction of remaining TrXP concentration in the reaction mixture from that added initially, respectively. Although the degradation of TrCP, TrBP and TrIP was enhanced by the HQ-HA-FeTHP catalysts (Figure 2), the levels of dehalogenation were not dependent on the types of catalysts and solution pHs in all of the TrXPs. The order of halogen substituents for the dehalogenation number was F > Cl > Br > I, in agreement with the order of electronegativity for halogens.

### 2.2. Turnover Numbers for the Degradation and Dehalogenation of TrXPs

While the percentage of TrXP degradation did not vary substantially at pH 3 or the [catalysts] 5 μM (Figure 2), the degree of dehalogenation was dependent on the type of TrXP used. The efficiencies of TrXP degradation should be evaluated precisely in terms of moles of degraded substrate per mole of catalyst per minute, that is, as the turnover frequency. However, the TrXPs were degraded within 1 min and after this period, a plateau was reached for all catalysts at pH 3. Thus, it was not possible to evaluate the kinetics of degradation TrXPs in the present study and the efficiency of TrXP degradation was evaluated as moles of degraded substrate per mole of catalyst, that is, the turnover number (TON).

**Figure 4:**
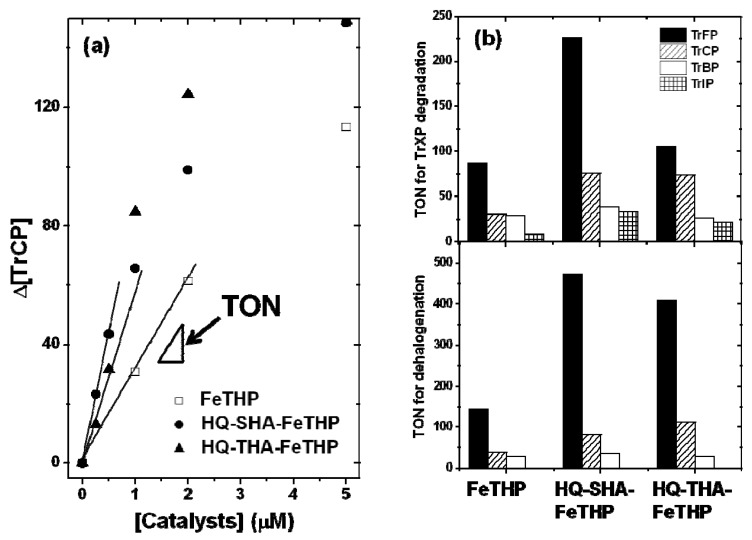
(**a**) Influence of catalyst concentrations on Δ[TrCP]; (**b**) Turnover numbers (TONs) for each catalyst. Conditions: pH 3, [TrXP] 150 μM, [KHSO_5_] 500 μM.

The TON values for each catalyst were calculated, based on the relationships between Δ[TrXP] and [catalyst], as shown in Figure 4a for the case of TrCP. Linear regions were observed for lower concentrations of catalysts, and the slope of the line corresponded to the TON values for each catalyst. These values indicate that a lower concentration of the catalyst results in higher levels of degradation or dehalogenation, *i.e.*, that the catalytic activity was high. For the efficiency of dehalogenation, the TON values indicate the molar numbers of released halide ions per mole of catalyst. Figure 4b shows the estimated TON values for TrXP degradation and dehalogenation using each catalyst. The TON values for TrFP in all catalysts were much larger than those for the other TrXPs, while the order of TON for TrXP degradation and dehalogenation was in general agreement with the order of electronegativity of the halogen substituents (F 4.10, Cl 2.83, Br 2.23, I 2.21). The higher TON values for TrFP may be attributed to the much higher electronegativity of F.

### 2.3. Oxidation Products

**Scheme 1:**
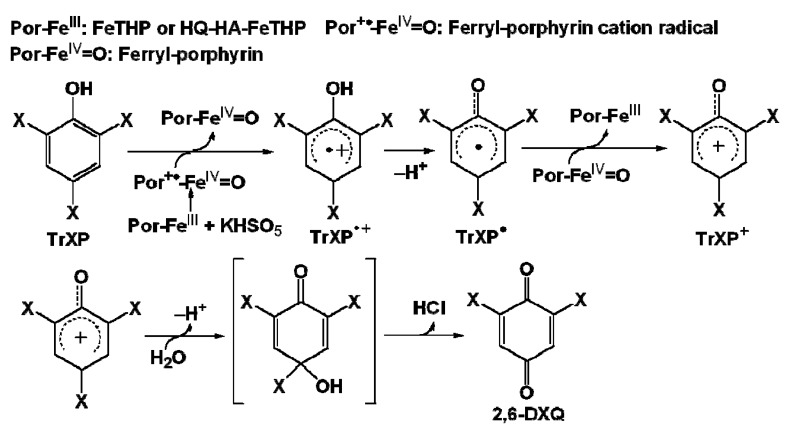
Oxidation pathways for producing 2,6-DXQ in the Fe(III)-porphyrin/KHSO_5_ catalytic system.

The electronegativity of the halogen substituents in the TrXPs can alter the nucleophilicity of their attached carbons. That is, the nucleophilicity of carbon would increase, if a halogen substituent with a higher electronegativity like F were attached. Scheme 1 illustrates the oxidation pathways for the production of a major oxidation product, 2,6-dihalogenated quinone (2,6-DXQ), by the Fe(III)-porphyrin/KHSO_5_ catalytic systems [5,19].

The production of 2,6-DXQ can be attributed to the addition of OH^−^ as a nucleophile from dissociated H_2_O to the nucleophilic carbon. If the trends in Figure 4b were responsible for the order of nucleophilicity of *para*-carbons, the levels of the produced 2,6-DXQ could parallel with the order of electronegativity of the halogen substituents on *para*-carbons. To examine this hypothesis, 2,6-DXQs in reaction mixtures at pH 3 were analyzed by GC/MS after extraction with *n*-hexane.

**Figure 5:**
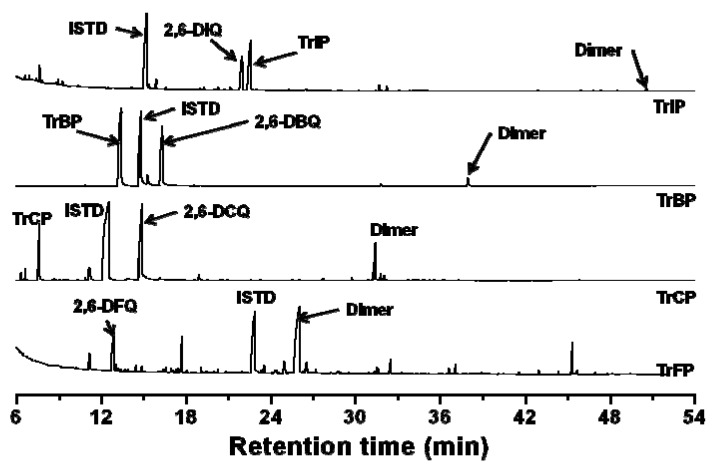
GC/MS chromatograms of hexane extracts of reaction mixtures using a HQ-SHA-FeTHP catalyst: [Catalyst] 5 μM, [TrXP]_0_ 150 μM, [KHSO_5_] 500 μM, pH 3. “ISTD” means internal standard (anthracene).

**Figure 6:**
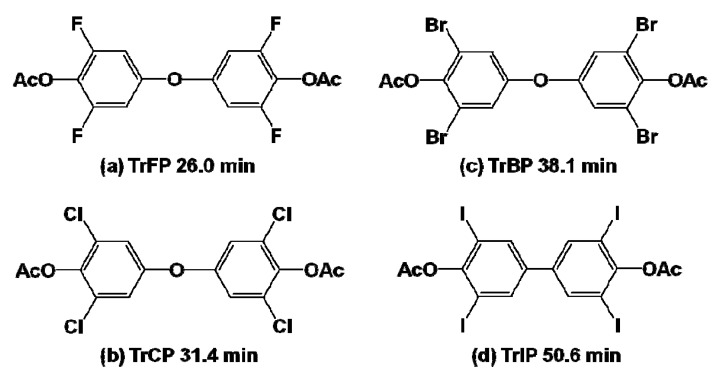
Major dimers produced from each TrXP.

Figure 5 shows the GC/MS chromatograms of hexane extracts from the reaction mixtures by the HQ-SHA-FeTHP/KHSO_5_ catalytic system. While 2,6-DXQs were detected as a result of the oxidation of TrXP, dimer products were also found. The structures of the dimers were identified, as shown in Figure 6, based on their mass spectra (*m*/*z* [fragment ions, relative intensity]): (a) 358 [M^+^, 1.46], 316 [(M − CH_2_CO)^+^, 41.9], 274 [(M − (CH_2_CO)_2_)^+^, 100]; (b) 424 [M^+^, 0.25], 382 [(M − CH_2_CO)^+^, 12.2], 340 [(M − (CH_2_CO)_2_)^+^, 53.3], 268 [(M − (CH_3_CO)_2_Cl_2_)^+^, 6.09]; (c) 602 [M^+^, 0.15], 560 [(M − CH_2_CO)^+^, 6.57], 518 [(M − (CH_2_CO)_2_)^+^, 26.6], 358 [(M − (CH_2_CO)_2_Br_2_)^+^, 6.89]; (d) 774 [M^+^, 0.36], 732 [(M − CH_2_CO)^+^, 12.1], 690 [(M − (CH_2_CO)_2_)^+^, 36.9], 562 [(M − (HI)(CH_2_CO)_2_)^+^, 1.56], 436 [(M − (CH_2_CO)_2_I_2_)^+^, 3.85].

**Figure 7:**
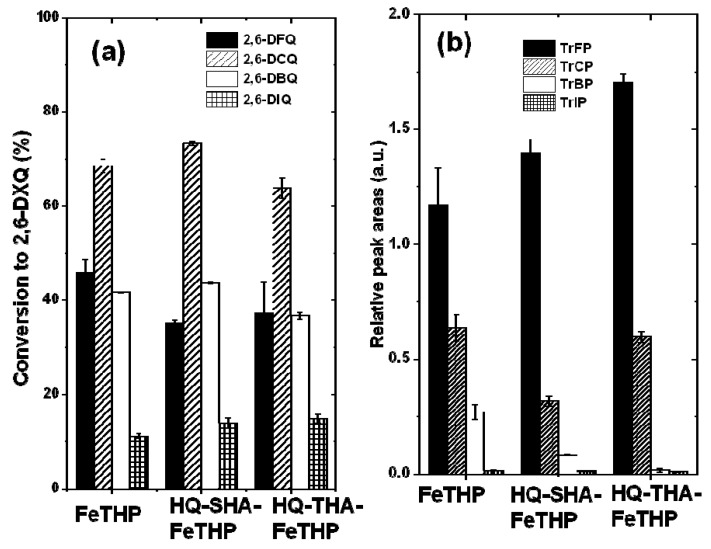
(**a**) Percent conversion of TrXPs to 2,6-DXQs; (**b**) Relative peak areas for the produced dimers.

Figure 7a shows the percent conversion of TrXPs to 2,6-DXQs in the reaction mixtures for each catalytic system. The levels of 2,6-DCQ conversion in all catalytic systems were larger than those for 2,6-DFQ, in contrast with the above mentioned hypothesis. However, the order of the relative peak areas for dimers from TrXPs, which were calculated by dividing the peak areas of dimers by that of anthracene, an internal standard (ISTD), was TrFP > TrCP > TrBP > TrIP (Figure 7b), consistent with the electronegativity of the halogen substituents. 

**Scheme 2:**
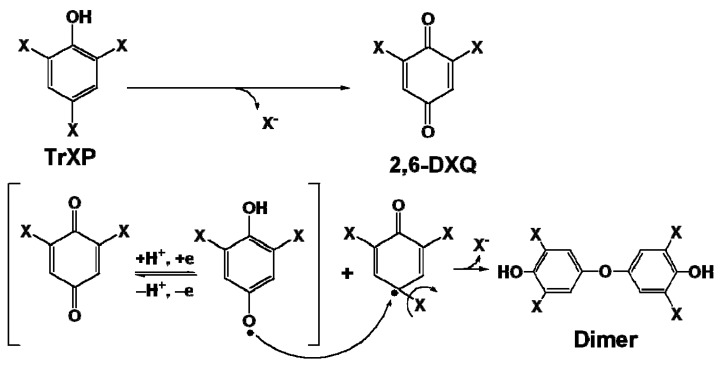
Formation of the dimer from TrXP and 2,6-DXQ via the radical coupling.

As shown in Scheme 2, semiquinone radicals can be produced in the reaction mixture due to the redox equilibrium of 2,6-DXQ. Such radical species are delocalized on aromatic rings, and this brings about the formation of a variety of coupling products [23,24]. Thus, TrXPs in the FeTHP/KHSO_5_ and HQ-HA-FeTHP/KHSO_5_ catalytic systems can finally be oxidized to coupling compounds such as the dimers in Figure 6 via 2,6-DXQs as reaction intermediates. As also shown in Scheme 2, because the conversion of 2,6-DFQ to a dimer can be facilitated in the case of TrFP, the levels of 2,6-DFQ are lower than those of 2,6-DCQ. The higher levels of dimer formation in the TrFP support the electronegativity of halogen substituents in TrXPs, an important factor that affects their oxidation efficiencies, such as the numbers of atoms that are removed by dehalogenation.

## 3. Experimental

### 3.1. Reagents and Materials

The FeTHP was synthesized as described in a previous report [19]. KHSO_5_ was obtained as a triple salt, 2KHSO_5_·KHSO_4_·K_2_SO_4_ (Merck). TrFP, TrCP, TrBP and TrIP were purchased from Aldrich, and a stock solution (0.01 M) was prepared in acetonitrile. 2,6-DCQ was purchased from Tokyo Chemical Industry Co. Ltd., and other 2,6-DXQs were synthesized according to a previous report [25]. HA samples were extracted from two soil samples [Shinshinotsu peat soil (SHA) and Tohro ando soil (THA)] and purified by a method approved by the International Humic Substances Society [20]. 

### 3.2. Synthesis of HQ-HA-FeTHP

**Scheme 3:**
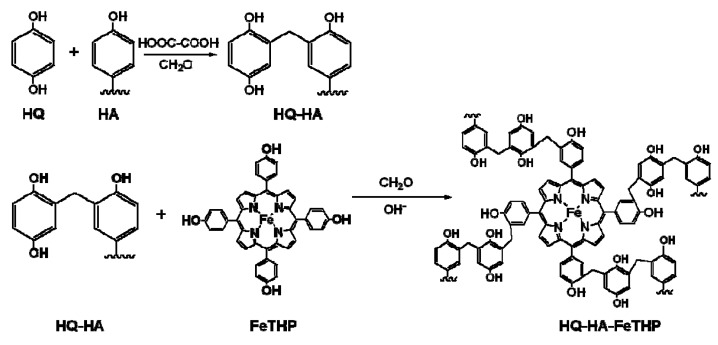
Synthesis of the HQ-HA-FeTHP catalysts.

The HQ-HA-FeTHP catalysts were synthesized, as shown in Scheme 3. A weighed sample of powdered HA (0.5 g, phenolic hydroxyl groups: 6.73 mmol for SHA; 5.50 mmol for THA) was placed in a 200-mL beaker and dissolved in 0.2 M NaOH aqueous (50 mL) under an atmosphere of N_2_. After neutralization with aqueous H_2_SO_4_, *p*-hydroquinone (0.4 g, 7.3 mmol of phenolic hydroxyl groups) was added and dissolved with vigorous stirring. After adding oxalic acid (50 mg) and 37% aqueous formaldehyde (0.5 g, 6.11 mmol), the mixture was transferred to a 300-mL round-bottom flask and the solution was refluxed for 1.0 h. The reaction mixture was then transferred to a 300-mL beaker and cooled in an ice-bath. After acidification with aqueous H_2_SO_4_ to pH 1, the resulting *p*-hydroquinone-derived HA (HQ-HA) precipitate was separated by centrifugation. The HQ-HA was purified by dialysis (500 Da) and a powdered sample was then obtained by freeze-drying. Subsequently, weighed 30 mg portions of HQ-HA and FeTHP powder were placed in a 300-mL round-bottom flask and dissolved in aqueous NaOH (60 mL, 0.1 M) under an atmosphere of N_2_. After adding formaldehyde (4.4 mmol), 5% aqueous NaOH (15 mL) was added with vigorous stirring, and the aqueous mixture was refluxed under a N_2_ atmosphere for 2 h. The cooled reaction mixture was then neutralized with aqueous H_2_SO_4_ and the solution concentrated and roughly deionized by an ultrafiltration technique (1,000 Da). After dialysis against pure water, a powdered sample was obtained by freeze-drying. The prepared HQ-HA-FeTHP catalysts with SHA and THA are abbreviated as HQ-SHA-FeTHP and HQ-THA-FeTHP, respectively. The molar contents of the prepared catalysts were estimated by determining the iron content in the prepared samples.

A weighed 1–2 mg portion of the powdered sample was placed in a glass vial and dissolved in a small amount of aqueous 0.01 M NaOH. An aliquot of an aqueous solution of H_2_O_2_ (1 mL, 33%) was added and the catalyst was then subjected to an ultrasonic treatment. The solution was diluted to 10 mL with a 0.1 M HCl solution. The iron in this solution was determined by ICP-AES (SPS7800-type, SII NanoTechnology, Inc.). The elemental compositions (C, H, N, O, S and ash contents, wt%) and iron contents of the synthesized HQ-HA-FeTHP catalysts are summarized in Table 1. Other spectroscopic data for HQ-HA-FeTHP catalysts (FT-IR and UV-Vis absorption spectra) can be found in a previous report [20].

**Table 1:** Elemental composition and iron content of the synthesized HQ-HA-FHP catalysts.

Catalyst	Elemental composition (wt %)						Fe content (μmol mg^−1^)
	C	H	N	O	S	ash	
HQ-SHA-FeTHP	60.00	5.30	4.31	23.99	0.39	6.01	0.55
HQ-THA-FeTHP	58.66	4.71	4.02	26.15	0.94	5.52	0.60

### 3.3. Tests for Catalytic Activities

An aliquot of buffer solution (2-mL, pH 3–7) was placed in a 20-mL L-shaped test tube. An aliquot of 0.01 M TrXP (30μL) in acetonitrile and an aliquot of an aqueous catalyst solution (50 μL, 200 μM) were then added to the buffer solution. Subsequently, 0.1 M aqueous KHSO_5_ (10 μL) was added, and the test tube was shaken in a Monosin IIA-type thermostatic shaking water bath (TAITEC) at 25 ± 0.1 °C. After a 30-min reaction period of shaking, 2-propanol (1 mL) was added to the solution. To analyze TrXP in the reaction mixture, an aliquot (20 μL) was injected into a PU-980 type HPLC pumping system (Japan Spectroscopic Co., Ltd.). The mobile phase consisted of a mixture of aqueous H_3_PO_4_ and methanol, and mixing ratios (H_3_PO_4_/methanol = v/v) were as follows: 40/60 for TrFP; 22/78 for TrCP; 20/80 for TrBP; 17/83 for TrIP. The flow rate was set at 1 mL min^−1^. The detection wavelengths of TrXP were set as follows: 220 nm for TrCP, TrBP and TrIP; 210 nm for TrFP. A 5C18-MS Cosmosil packed column (4.6 mm i.d. × 250 mm, Nacalai Tesque) was used as the solid phase, and the column temperature was maintained at 50 °C. After analyzing the TrXP, the concentrations of halide ions (F^−^, Cl^−^, Br^−^ and I^−^) released during the catalytic oxidation were determined by ion chromatography (IC-20 type, Dionex). An AS-12 type column with a mixture of 2.7 mM Na_2_CO_3_ and 0.3 mM NaHCO_3_ as an eluent was employed for the F^−^, Cl^−^ and Br^−^ analyses, and an AS-16 type column with the eluent of 35 mM NaOH aqueous was employed for I^-^ analysis.

The byproducts produced during the catalytic oxidation of TrXP were identified by means of a GC/MS technique after extracting the reaction mixture with *n*-hexane. The catalytic oxidation system, described above, was scaled up to 25 mL at pH 3. After reaction periods of 30-min, a 1 M aqueous solution of ascorbic acid (1 mL) was added, and the pH of the solution was adjusted to 11–11.5 by adding aqueous K_2_CO_3_ (600 g L^−1^). Subsequently, acetic anhydride (5 mL) was added dropwise to the solution, and a 1 mM anthracene solution in hexane (0.5 mL) was added as an internal standard (ISTD) for the GC/MS analysis. This mixture was extracted with 20 mL portions of *n*-hexane, and the combined extract was then dried over anhydrous Na_2_SO_4_. After filtration, the extract was evaporated under a stream of dry N_2_, and the residue was dissolved in *n*-hexane (0.25 mL). An aliquot of the extract (1 μL) was introduced into a GC-17A/QP5050 GC/MS system (Shimadzu). A Quadrex methyl silicon capillary column (0.25 mm i.d. × 25 m) was employed in the separation. The temperature ramp was as follows: 65 °C for 1.5 min, 65–120 °C at 35 °C min^−1^, 120–300 °C at 4 °C min^−1^ and a 300 °C held for 10 min for the cases of TrCP, TrBP and TrIP; 65 °C for 1.5 min, 65–300 °C at 5 °C min^−1^, 300 °C held for 4 min for TrFP.

## 4. Conclusions

The influence of halogen substituents in TrXPs on the characteristics of their oxidation by FeTHP/KHSO_5_ and HQ-HA-FeTHP/KHSO_5_ catalytic systems was examined. While the HQ-HA-FeTHP catalysts were effective in the dehalogenation of TrFP and TrCP, TrBP and TrIP were dehalogenated less easily by both the FeTHP and HQ-HA-FeTHP systems. The order of halogen substituents in TrXP for the oxidative dehalogenation was F > Cl > Br > I, consistent with the order of electronegativity of halogens. A higher electronegativity of a halogen substituent can lead to a higher nucleophilicity of the attached carbon, and this facilitates the attack of a nucleophile such as OH^-^ in aqueous solutions to form oxidation products, such as 2,6-DXQs and dimers. Although the levels of 2,6-DXQ formed as a result of oxidation could be one of the indices for the trend for the nucleophilic addition of OH^-^, which accompanies the dehalogenation, the levels of 2,6-DFQ were lower than those for 2,6-DCQ, contrary to the above expectations. However, the levels of dimer from TrFP were much larger than the corresponding values for the other TrXPs. This supports the conclusion that dimers from TrXPs are formed via 2,6-DXQ as reaction intermediates. These results led to a conclusion that the trend for the dehalogenation for TrXPs via the iron(III)-porphyrin/KHSO_5_ catalytic systems is dependent on the electronegativity of their halogen substituents.
